# Response to Brenaut et al., “Response to Pham et al’s ‘Sensory symptoms of scalp psoriasis are not sensitive scalp syndrome’”

**DOI:** 10.1016/j.jdin.2026.02.002

**Published:** 2026-02-17

**Authors:** Nguyen Pham, Chuyen Thi Hong Nguyen, Trung The Van

**Affiliations:** Department of Dermatology, University of Medicine and Pharmacy, Ho Chi Minh City, Vietnam

**Keywords:** psoriasis, scalp psoriasis, sensitive

To the Editor,

We thank Brenaut et al^1^ for their careful reading of our work^2^ and for their thoughtful comments. We welcome this opportunity to clarify the conceptual scope of our study.

Our investigation was prompted by a recurring clinical observation: some patients with scalp psoriasis report prominent sensory symptoms that appear disproportionate to the visible severity of their disease, whereas others with more extensive involvement describe minimal discomfort. Such discordance may contribute to the underrecognition of symptom burden in routine practice and may influence clinical evaluation. Our intention was therefore to examine sensory manifestations within this specific clinical context, rather than to reinterpret established definitions or challenge the existing nosologic framework.

We fully agree that idiopathic sensitive skin should be distinguished from sensitivity associated with an underlying dermatosis.^3^ In this regard, the term “psoriasis-associated scalp sensitivity” may better characterize our cohort, reflecting a secondary form of scalp sensitivity rather than an idiopathic condition.^4^^,^^5^ Explicit recognition of this distinction helps preserve conceptual clarity while highlighting sensory symptoms that may carry meaningful clinical implications in inflammatory scalp disorders.

We appreciate these constructive remarks, which underscore the importance of continued research to refine terminology and further elucidate underlying mechanisms. We hope that our findings contribute, in a complementary manner, to a more nuanced and patient-centered understanding of scalp disease.

## Conflicts of interest

None disclosed.
